# Changes in mean systemic filling pressure as an estimate of hemodynamic response to anesthesia induction using propofol

**DOI:** 10.1186/s12871-022-01773-8

**Published:** 2022-07-22

**Authors:** Maayan Zucker, Gregory Kagan, Nimrod Adi, Ilai Ronel, Idit Matot, Lilach Zac, Or Goren

**Affiliations:** 1 Sackler Faculty of Medicine, Tel Aviv University, Tel Aviv, Israel; 2 Division of Anesthesiology, Pain, and Intensive Care, Tel Aviv Medical Center, Sackler Faculty of Medicine, Tel Aviv University, Tel Aviv, Israel

**Keywords:** Cardiac output, General anesthesia, Mean systemic filling pressure, Propofol, Venous resistance

## Abstract

**Background:**

Even a small change in the pressure gradient between the venous system and the right atrium can have significant hemodynamic effects. Mean systemic filling pressure (MSFP) is the driving force of the venous system. As a result, MSFP has a significant effect on cardiac output. We aimed to test the hypothesis that the hemodynamic instability during induction of general anesthesia by intravenous propofol administration is caused by changes in MSFP.

**Methods:**

We prospectively collected data from 15 patients undergoing major surgery requiring invasive hemodynamic monitoring. Hemodynamic parameters, including MSFP, were measured before and after propofol administration and following intubation, using venous return curves at a no-flow state induced by a pneumatic tourniquet.

**Results:**

A significant decrease in MSFP was observed in all study patients after propofol administration (median (IQR) pressure 17 (9) mmHg compared with 25 (7) before propofol administration, *p* = 0.001). The pressure gradient for venous return (MSFP – central venous pressure; CVP) also decreased following propofol administration from 19 (8) to 12 (6) mmHg, *p* = 0.001. Central venous pressure did not change.

**Conclusions:**

These results support the hypothesis that induction of anesthesia with propofol causes a marked reduction in MSFP. A possible mechanism of propofol-induced hypotension is reduction in preload due to a decrease in the venous vasomotor tone.

**Supplementary Information:**

The online version contains supplementary material available at 10.1186/s12871-022-01773-8.

## Background

Preload and cardiac output (CO) are tightly regulated by the maintenance of a pressure gradient between the low-pressure venous system and the right atrium [1]. Mean Systemic Filling pressure (MSFP) is a theoretical term which describes the pressure in the systemic vascular system in a no-flow state, and is estimated at about 7–10 mmHg [2]. MSFP depends on the stressed volume (the volume in the venous system generating pressure), the venous capacitance, and venous compliance [3]. Mean Circulatory Filling pressure is defined as the pressure that exist in the whole circulation when the heart seized to work, and it can be measured using the Parm method [4]. Measuring MSFP is almost impossible in living person who are not mechanically ventilated. Since the pulmonary circulation has less than one-eighth capacitance and one-tenth blood volume as the systemic circulation, the differences between MSFP and MCFP values are insignificant [1].

Approximately 70% of the blood volume is contained in the venous system, and compliance is about 30 times greater than in the arterial system, which allows the venous system to function as a reservoir [5]. Accordingly, MSFP is a good indicator of the venous system driving pressure. Venous return (VR) is determined by the difference between the MSFP and the right atrial pressure (P_RA_), divided by the venous resistance [1]. Changes in the pressure gradient or an increase in the venous resistance will affect venous return [6]. Since VR equals CO in equilibrium, a constant CO can be maintained by increasing the resistance to venous return (R_VR_), compensating for any increase in the MSFP-CVP gradient (pressure to venous return; P_VR_) [7, 8]. On the other hand, in a hypovolemic patient, passive leg raising or fluid administration will increase the MSFP-CVP gradient [9]. This increase in P_VR_ will increase the preload and contractility but with a smaller change in the VR, thus creating a new equilibrium of the system when VR equals CO again. Global cardiac efficiency (E_h_) is another parameter based on the MSFP providing an estimate of cardiac performance [10].

Propofol is the most common intravenous agent used to induce general anesthesia. It is known to cause hypotension [11, 12], with possible mechanisms including decrease in sympathetic tone [13, 14], a decrease in preload and afterload [15], or direct myocardial depression [16]. Aside of the pharmacological effects, the transition from spontaneous breathing to positive pressure ventilation affects both the venous return and the peripheral resistance [17].

Only few studies evaluated propofol’s effects on the venous system during anesthesia induction or maintenance [18–21]. However, none have evaluated specifically changes in the vasomotor tone of the venous system during induction of anesthesia with propofol. Measuring venous vasomotor effects during induction may isolate the hemodynamic changes occurring due to propofol administration from the effects of positive pressure ventilation [22].

We therefore aimed to characterize changes in hemodynamic parameters of the venous system during induction of general anesthesia by propofol, in order to better describe the underlying mechanism of propofol-induced hypotension. Specifically, we aimed to measure changes in MSFP and CVP and their effect on venous return and cardiac output.

## Methods

### Patients

We conducted a prospective observational single-center study between January and October 2019. Ethical approval (0393–18 TLV) was granted by the institutional review board of the Tel-Aviv Sourasky medical center, Tel Aviv, Israel on the 23/07/2018. Written informed consent was obtained from all subjects prior to enrollment.

The study enrolled patients 18 years or older, with American Association of Anesthesiologists (ASA) physical status score I-III, scheduled for major elective surgery that dictated the use of invasive hemodynamic monitoring including CVP (measured by a central venous catheter inserted through the internal jugular to the superior vena cava near the right atrium) and invasive continuous arterial blood pressure monitoring.

Patients with heart failure (New York Heart Association (NYHA) classification grade 3 or more), moderate to severe pulmonary hypertension, end-stage renal disease, or peripheral vascular disease were excluded. Patients appointed to surgeries involving major vessels such as vena cava, right atrium, etc.; cases involving hemodynamically compromised patients due to mass effect or pressure on major vessels and cases involving combined multidisciplinary teams (such as general surgery and cardiac surgery teams) were also excluded.

### Measurements

Prior to surgery, a radial artery cannulation, central venous line, and an antecubital fossa venous line were inserted under a continuous infusion of remifentanil at a starting dose of 0.05 mcg·min·kg^− 1^ titrated to light sedation. The arterial and peripheral venous lines were inserted in the same arm, and all lines were connected to pressure transducers located close at the mid-axillary line, in level with the phlebostatic line. The CVP transducer was placed close to the fourth intercostal space. Patients were supine during the whole study protocol, and the hand with the arterial and venous cannulations was in level with the arterial and venous transducers.

Approximately half the cases required epidural anesthesia, which was conducted before the commencement of anesthesia. A test dose and/or a therapeutic dose of local anesthetics were injected only after the hemodynamic measurements were taken. None of the cases required peripheral nerve blocks.

Remifentanil was discontinued immediately after lines insertion and before MSFP measurements. After patients regained full consciousness, a first MSFP measurement (pre-induction) was conducted. The patients were then pre-oxygenated using a fraction of inspired oxygen (FiO_2_) of 1. An induction dose of 2 mg/kg propofol bolus was administered, followed by a second MSFP measurement (post-induction) which was aimed to be measured when we assumed the propofol effect was maximal (the BP value was the lowest). Endotracheal intubation was facilitated by administration of 1 mcg/kg of fentanyl and 0.6 mg/kg of rocuronium. A third MSFP measurement (post-intubation) was performed 2 min after intubation. Ventilation parameters were unified during the study, including positive end expiratory pressure (PEEP) values of 5 mmHg, respiratory rate of 12 per minute and tidal volume calculated as 7ccXkg of ideal body weight.

Each measurement included systolic, diastolic, and mean (MAP) arterial blood pressures, heart rate, CVP, peripheral venous pressure, CO, and MSFP recordings. All values were recorded from the monitor in real-time in our case report form (CRF).

MSFP was measured using the Parm method, which was described elsewhere [4]. A surgical pneumatic tourniquet was placed around the upper arm, ipsilateral to the arterial and venous lines. The tourniquet was inflated to a pressure of at least 100 mmHg above the systolic blood pressure and no less than 250 mmHg to a maximal duration of 60 seconds. Within those 60 seconds, MSFP was measured at the point where the arterial and venous pressures equalized, and a no-flow state was reached. Cardiac output was measured using the FloTrac™ system (Edwards Lifesciences Crop., California, USA).

### Data analysis and statistics

Systemic vascular resistance (R_sys_) was determined by the dividing the pressure gradient between the aortic blood pressure (P_AO_) and the right atrial pressure (P_RA_) by the venous return (VR). P_AO_, P_RA_, and VR were represented by MAP, CVP, and CO, respectively.

$${R}_{sys}=\frac{P_{AO}-{P}_{RA}}{VR}=\frac{MAP- CVP}{CO}$$

Resistance to venous return (R_VR_) was calculated using the following equation, where *P*_RA_ and VR are represented by CVP and CO, respectively:

$${R}_{VR}=\frac{MSFP-{P}_{RA}}{VR}$$

Global cardiac efficiency (E_h_) was calculated using the following equation:

$$Eh=\frac{MSFP-{P}_{RA}}{MSFP}$$

To the best of our knowledge, measurements of MSFP during the induction of anesthesia were never performed in humans. However, we have conducted a post-hoc justification of power based on a similar study conducted by de Wit and colleagues [20]. Using the WINPEPI software ver.11.65, we have calculated the sample size needed to find a difference of 6.5 mmHg in the MSFP results. Using a sample size formula for paired measures (assuming a two-sided α of 5%), we found the power of our study to be 93%.

Continuous variables are reported as median and interquartile range (IQR). Categorical variables are reported as incidence and percentage. Statistical significance was tested using the Wilcoxon test, a non-parametric test designed to analyze paired or repeated measures, when assumptions for normal distribution are not met. A *P*-value of < 0.05 was considered significant.

## Results

Eighteen patients were enrolled; three were excluded from the study due to surgery cancellation or delay. Of the remaining 15, 6 were females, with median age of 66 (range 40–76) years. Demographic and baseline characteristics are detailed in Table 1. All patients underwent elective major abdominal surgery.

**Table 1:** Patient demographic and baseline characteristics

	All Patients
Age, years	66 (40–76)
Sex, n (%)	
Male	9 (60)
Female	6 (40)
Height, cm	170 (15)
Weight, kg	79 (22)
Body mass index (kg m^−2^)	27 (7)
ASA score	2 (1)
Cardiovascular diseases, n (%)	
Ischemic heart disease	1 (7)
Arrhythmia	1 (7)
Hypertension	5 (33)
Baseline medications, n (%)	
Beta blockers	3 (20)
Alpha blockers	1 (7)
ACE inhibitors	3 (20)
Angiotensin receptor blockers	2 (13)
Calcium channel blockers	3 (20)
Thiazides	3 (20)
Spironolactone	1 (7)
Anesthesia type, n (%)	
General	7 (47)
Combined general + regional	8 (53)

Values are presented as median (range), median (interquartile range), or n (%), as appropriate

*ASA* American Society of Anesthesiologists physical status score

The measurements recorded before induction with propofol, following induction, and 2 min after intubation are presented in Table 2. Median (IQR) MSFP values decreased significantly from 25 (7) mmHg pre-induction to 17 (9) mmHg after induction, *P* = 0.001. MSFP significantly increased after intubation (25 (9) mmHg, *P* = 0.003 compared to post-induction values). Of note, CVP values did not show a clinically important decrease before and after induction, with a mean (SD), decrease of 0.4 (3.5) mmHg. P_VR_ decreased from a median baseline of 19 (8) to 12 (6) mmHg after induction (*P* = 0.001) and increased to 19 (7) after intubation; (*P* = 0.001 compared to pre-induction values).

**Table 2:** Hemodynamic measurements before induction, after induction, and after intubation

Parameter	Pre induction	Post induction		Post Intubation	
	Median (IQR)	Median (IQR)	P_**a**_^**†**^	Median (IQR)	P_**b**_^**‡**^
HR (beats × min^−1^)	82 (17)	77 (16)	0.004	90 (27)	0.002
MAP (mmHg)	100 (11)	78 (27)	0.001	89 (29)	0.025
CVP (mmHg)	7 (8.5)	5 (7.5)	0.590	9 (4.5)	0.026
MSFP (mmHg)	25 (7)	17 (9)	0.001	25 (9)	0.003
MSFP-CVP (mmHg)	19 (8)	12 (6)	0.001	19 (7)	0.001
CO (L × min^−1^)	6.1 (1.6)	4.8 (2.3)	0.002	5 (2.1)	0.798
R_sys_ (mmHg × min × L^−1^)	14.3 (3)	14.7 (6)	0.233	15 (4)	0.156
R_VR_ (mmHg × min × L^−1^)	3.1 (2.5)	2.2 (2.2)	0.069	3.3 (1.9)	0.011
E_h_	0.73 (0.24)	0.67 (0.35)	0.094	0.70 (0.19)	0.91

*IQR* interquartile range, *HR* heart rate, *MAP* mean arterial pressure, *CVP* central venous pressure, *MSFP* mean systemic filling pressure, *P*_*VR*_ pressure gradient of venous return (MSFP-CVP), *CO* cardiac output, *R*_*sys*_ systemic vascular resistance, *R*_*VR*_ resistance to venous return, *E*_*h*_ cardiac efficiency

^†^P_a,_ Wilcoxon test between pre induction values to post induction values

^‡^P_b,_ Wilcoxon test between post induction values to post intubation values

**Fig. 1:**
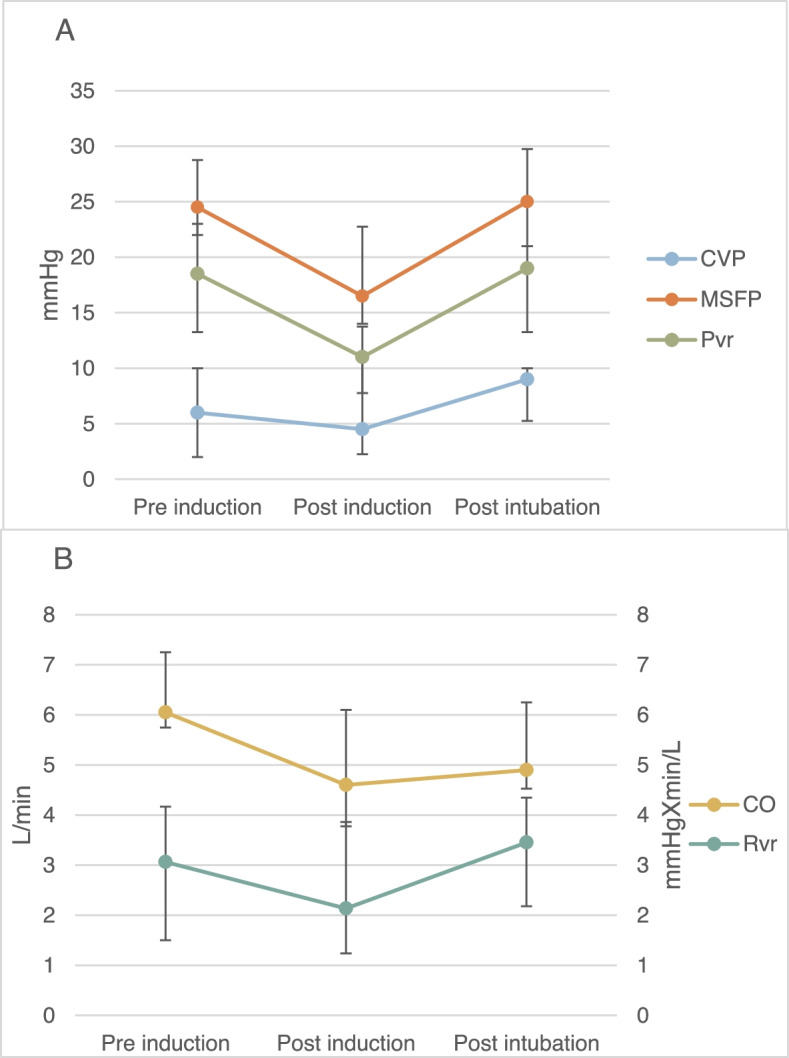
**A** Changes in mean systemic filling pressure, central venous pressure, and pressure gradient to venous return; **B** Changes in cardiac output and resistance to venous return. Changes in mean systemic filling pressure (MSFP), central venous pressure (CVP) pressure to venous return (P_VR_), cardiac output (CO) and resistance to venous return (R_VR)_ throughout induction of anesthesia

CO decreased from 6.1 (1.6) L/min before induction to 4.8 (2.3) L/min after induction; *P* = 0.002. R_sys_, R_VR_ and E_h_ did not change significantly after propofol administration. The effects of propofol administration on MSFP, CVP, P_VR_, CO and *R*_*VR*_ are presented in Fig. 1.

**Fig. 2:**
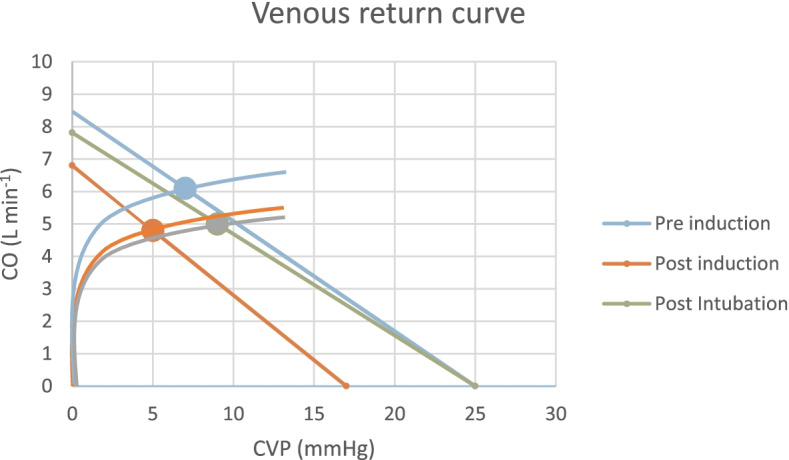
Venous return and cardiac output curves. Venous return and cardiac output curves for pre-induction, post-induction and post intubation measurements that were constructed using mean values of cardiac output (CO), central venous pressure (CVP) and mean systemic filling pressure

An estimation of the venous return curves using CO, CVP, and MSFP values is presented in Fig. 2.

According to medical records, 33% of the patients had hypertension and 47% were treated with antihypertensive drugs, but only 20% of them took antihypertensive medication in the 24 h prior to surgery (one patient used beta blocker [BB], one patient used calcium channel blocker [CCB], and one patient used angiotensin receptor blocker [ARB]). We conducted statistical analysis comparing the group with a medical record of hypertensive drugs use with the group without such record. We had then conducted a sub-analysis of our study population again, comparing the three patients who used these drugs 24 hours before surgery with those who did not use antihypertensive drugs in those 24 hours. We analyzed all the main hemodynamic parameters using the non-parametric Mann Whitney test and found no significant statistical difference between these groups, the analysis is presented in Table 1 in the Supplementary data.

## Discussion

Our results show that induction of anesthesia using propofol causes a significant reduction in MSFP. Similar effects were evident on the pressure gradient of venous return (P_VR_) and the CO. In contrast, CVP did not decrease significantly during induction, as seen in Fig. 1. These results support our hypothesis that induction of anesthesia using propofol has significant effects on the venous system as a whole, and MSFP in particular. Figure 2 illustrates the pre- and post-induction venous return curves and shows the post-induction shift clockwise. However, unlike the results of de Wit and colleagues [20], we observed a decrease in CO in the post-induction venous return curve. This effect might be explained by the difference in propofol administration between the two studies; our use of bolus instead of continuous administration had a more dramatic effect on the CO. When venous pressure decreases, CO will consequently decrease if the change is immediate and the cardiovascular system does not have enough time to compensate, as in bolus administration.

Unlike hypovolemia (causing a decrease in both CVP and MSFP), the observed reduction of CO in our study is probably the reason for the nonsignificant change in CVP. When a low E_h_ is dominant, MSFP and R_VR_ will decrease with a less marked change in CVP.

We would have expected a reduction in R_VR_ duo to propofol bolus administration. However, according to our observations, R_VR_ trended towards a decrease (from 3.1 $\mathsf{mmHg}\times \mathsf{\min}\times {\mathsf{L}}^{-\mathsf{1}}$ pre induction to 2.2 $\mathsf{mmHg}\times \mathsf{\min}\times {\mathsf{L}}^{-\mathsf{1}}$ post-induction, with only a marginal significance (*p* = 0.069)). Since R_VR_ is close to significance, we can only assume that a larger sample size could show a significant reduction. Post-hoc power justification of our study found it to be well powered to detect changes in MSFP. However, it may not be powered to detect changes in R_VR_.

Following propofol induction, cardiac output was significantly reduced, and cardiac efficiency demonstrated a trend of reduction, but it was not statistically significant. Global cardiac efficiency (E_h_) is a dimensionless ratio (0 < E_h_ < 1) and equals the difference between MSFP and P_RA_ divided by MSFP. E_h_ may be used as an estimate of inotropic function. For example, E_h_ approaches zero when cardiac output seizes and P_RA_ equals MSFP. The change we observed in cardiac efficiency was mostly proportional to the change in MSFP, while the change in CO originated from two contradicting mechanisms: reduced P_VR_ (decreasing VR and CO) and reduced R_VR_ (increasing VR and CO).

Intubation resulted in increase in MSFP, MAP and HR, but there was no change in CO. Our interpretation of those results is that as positive pressure ventilation increases P_RA_, MSFP will rise to maintain the driving pressure, but the rise in R_VR_ will decrease the VR and, consequently, decrease the stroke volume. CO remained stable due to the increase in HR. The increase in HR may be compensatory to the decrease in preload, response to the stress of intubation, or a combination of both.

The decrease in MAP after propofol administration for induction of anesthesia has been previously demonstrated [12, 15]. Other studies evaluated the effect of propofol infusion on MSFP and found a correlation between increasing dosages of propofol and MSFP reduction [20]. Our results show a significant and substantial negative effect of an induction dose on MSFP. In contrast to de Wit and colleagues [20], our results exhibit CO reduction in response to propofol administration. As we examined the effect of induction and not maintenance of anesthesia, this discrepancy can possibly be explained by the different administration methods (bolus vs. infusion), or the different doses.

Although the negative hemodynamic effects of propofol are well known, recent studies, including the current one, endeavoured to evaluate potential mechanisms involving the venous system. MSFP is a genuine indicator of the venous system; however, due to its infrequent use in clinical practice, the potential changes in response to hypnotic drugs such as propofol have been tested on humans scarcely. While analysing our results together with the study by de Wit and colleagues [20], we can conclude that the hemodynamic deterioration following propofol bolus administration is mediated to a large extent through its effect on the venous system. This effect is most likely regulated through the alteration in venous tone, shifting the venous volume from a stressed to an unstressed volume [22].

### Clinical implications

As the venous system, and the stressed volume in particular, constitute a key component in post-induction hypotension, propofol administration should be carried out carefully in hypovolaemic patients. Furthermore, the management of hemodynamic deterioration after propofol administration in a fluid-responsive patient may benefit from the use of vasoactive drugs alongside fluid loading in order to increase the stressed volume and the MSFP.

### Study limitations

This was a pilot study, and as such, we chose to adhere to a strict protocol of induction in relatively healthy and hemodynamically patients. Aside of two patients who received a 200 ml fluid bolus prior to induction, the drug administration was identical in all patients. We were therefore able to isolate the effect of propofol by evaluating the pre-induction and post-induction measurements with no additional influences, such as other hypnotic or vasoactive drugs or positive-pressure ventilation. Our results are therefore of limited generalizability to other patient populations or clinical situations, such as patients with active cardiovascular disease or hemodynamic instability. It is plausible that propofol administration has a more profound effect on patients with initially lower MSFP values.

The average age of the study population was correlated with high prevalence of hypertension, and antihypertensive medications, including BB, CCB, ARBs, thiazides and ACE inhibitors can influence CO in several mechanisms. Since our study focused on the CO change from baseline values rather than the absolute value, we assumed that the use of hypertensive drugs will not influence our results significantly. Nevertheless, statistical analysis conducted for this sub-group showed no statistically significant difference in their hemodynamic parameters.

Another possible limitation is the difficulty in evaluating the influence of propofol on cardiac contractility. Since it was not feasible to maintain a constant VR throughout the experiment, we cannot comment on the precise effects of propofol administration on cardiac contractility.

Due to practical limitations, our attempt to reduce patient inconvenience, and the short time interval between induction and tracheal intubation, we only acquired a single MSFP measurement in each time period. Nonetheless, repeated measurements of MSFP in other studies showed no significant difference, with a coefficient variance for a single measurement of 5% [4].

Future studies are necessary, and should include larger cohorts, preferably also evaluating hemodynamically unstable patients. Additionally, obtaining more MSFP measurements before and after inotropes administration may shed more light on mechanisms affecting MSFP.

## Conclusions

Induction of anesthesia using a bolus dose of propofol causes hemodynamic deterioration, evidenced by a decrease in MAP that is largely mediated by reduction in MSFP and its effects on the pressure gradient of venous return, which reflects its main effect on the venous system. The use of propofol bolus during induction of anesthesia in hemodynamically unstable patient should be avoided if possible.

## Supplementary Information

**Additional file 1: Table 1.** Sub-analysis for patients receiving antihypertensive drugs

## Data Availability

The datasets generated and/or analysed during the current study are not publicly available due to hospital’s policy regarding public data sharing of patients’ medical information, but are available from the corresponding author on reasonable request.

## Abbreviations

ACE: Angiotensin converting enzyme
ARB: Angiotensin receptor blocker
ASA: Association of anesthesiologists
BB: Beta blocker
BMI: Body mass index
CCB: Calcium channel blocker
CO: Cardiac output
CVP: Central venous pressure
E_h_: Cardiac efficiency
FiO_2_: Fraction of inspired oxygen
HR: Heart rate
HTN: Hypertension
IHD: Ischemic heart disease
IQR: Interquartile range
MAP: Mean arterial pressure
MCFP: Mean circulatory filling pressure
MSFP: Mean systemic filling pressure
NYAH: New York heart association
PEEP: Positive end expiratory pressure
P_RA_: Right atrial pressure
P_VR_: Pressure gradient of venous return
RAP: Right atrial pressure
R_sys_: Systemic vascular resistance
SD: Standard deviation
VR: Venous return
