# Supplementary material for: Changes in mean systemic filling pressure as an estimate of hemodynamic response to anesthesia induction using propofol
Source: BMC Anesthesiol. 2022 Jul 22;22:234. doi: 10.1186/s12871-022-01773-8 (PMC9306094; doi:10.1186/s12871-022-01773-8)
Supplement: Supplementary file 1 — Additional file 1: Table 1. Sub-analysis for patients receiving antihypertensive drugs [file 12871_2022_1773_MOESM1_ESM.docx]

**Table 1- Sub-analysis for patients receiving antihypertensive drugs**

| Antihypertensive drugs 24 hours before surgery | | | Antihypertensive drugs in medical records | | |  | |
| --- | --- | --- | --- | --- | --- | --- | --- |
| Pv | Antihypertensive drugs used in 24 hours  N=3 *  Median  * (IQR could not be calculated) | No antihypertensive drugs used in 24 hours  N=12  Median  (IQR) | Pv | No antihypertensive drugs in medical records  N=8  Median  (IQR) | Antihypertensive drugs in medical records  N=7  Median (IQR) |  |  |
| 0.45 | 93 | 100 (95-108) | 0.61 | 101 (92-110) | 97 (93-106) | MAP mmHg | Pre-Induction |
| 0.23 | 79 | 84 (77-98) | 0.61 | 88 (77-98) | 80 (74-85) | HR beats/min |  |
| 1.00 | 7.0 | 6.0 (0.5-13.0) | 0.23 | 8.5 (2.8-14.8) | 3.0 (0.0-10.0) | CVP mmHg |  |
| 0.37 | 11.0 | 13.0 (3.0-14.0) | 0.54 | 13 (5-14) | 11.0 (2.0-14.0) | Peripheral venous pressure mmHg |  |
| 0.45 | 25.0 | 24.5 (22.0-29.0) | 0.78 | 24.5 (22.0-28.8) | 25.0 (18.0-34.0) | MSFP mmHg |  |
| 0.73 | 6.0 | 6.2 (5.8-8.6) | 0.15 | 6.8 (6.0-9.4) | 6.0 (5.4-7.1) | CO L/min |  |
| 0.73 | 14.3 | 14.8 (10.4-18.0) | 0.40 | 14.2 (9.0-17.7) | 15.4 (14.0-16.9) | SVR mmHg*min/L |  |
| 0.95 | 0.71 | 0.75 (0.49-0.98) | 0.28 | 0.71 (0.42-0.88) | 0.72 (0.70-1.00) | Eh |  |
| 0.63 | 57 | 79 (62-86) | 0.34 | 81 (72-86) | 59 (54-90) | MAP mmHg | Post-Induction |
| 0.37 | 67 | 79 (70-88) | 0.19 | 84 (70-91) | 75 (64-80) | HR beats/min |  |
| 0.84 | 6.0 | 4.5 (2.3-12.8) | 0.40 | 6 (2.5- 13.5) | 4.0 (1.0-8.0) | CVP mmHg |  |
| 0.27 | 5.5 | 10.0 (4.5-15.3) | 0.40 | 10 (7-15) | 6 (2.0-15.3) | Peripheral venous pressure mmHg |  |
| 1.00 | 16.0 | 17.0 (14.0-23.8) | 0.69 | 17 (14.3-23.8) | 16.0 (12.0-26.0) | MSFP mmHg |  |
| 0.84 | 4.8 | 4.7 (3.3-8.0) | 0.28 | 5.6 (3.8-8.8) | 4.4 (2.9-6.1) | CO L/min |  |
| 0.64 | 12.7 | 15.1 (8.5-22.1) | 0.87 | 15.1 (6.4-20.9) | 12.7 (11.4-21.6) | SVR mmHg*min/L |  |
| 0.54 | 0.69 | 0.61 (0.44-0.86) | 0.23 | 0.58 (0.32-0.85) | 0.69 (0.56-0.92) | Eh |  |
| 0.84 | 97 | 87 (79-117) | 0.40 | 83 (79-101) | 102 (78-127) | MAP mmHg | Post-Intubation |
| 0.18 | 78 | 96 (77-108) | 0.34 | 101 (74-108) | 80 (76-94) | HR beats/min |  |
| 0.84 | 9.0 | 9.0 (5.3-10.8) | 1.00 | 9 (5.0-11.8) | 9.0 (6.0-10.0) | CVP mmHg |  |
| 0.28 | 9.0 | 11.0 (10.5-15.5) | 0.88 | 11 (10.5-12.5) | 11.0 (9.0-18.0) | Peripheral venous pressure mmHg |  |
| 0.63 | 29.0 | 25.0 (19.5-31.5) | 0.34 | 23 (19.5-29.5) | 29.0 (23.0-32.0) | MSFP mmHg |  |
| 0.84 | 5.0 | 5.3 (4.4-6.8) | 0.61 | 5.3 (4.6-7.9) | 5.0 (4.3-6.4) | CO L/min |  |
| 1.00 | 16.0 | 15.2 (12.1-19.7) | 0.46 | 14.4 (11.7-16.4) | 16.0 (12.6-20.8) | SVR mmHg*min/L |  |
| 0.63 | 0.70 | 0.71 (0.52-0.76) | 0.78 | 0.71 (0.50-0.80) | 0.70 (0.57-0.76) | Eh |  |

*This table represent a comparison between patients with medical record of hypertensive drugs use with the group without such record, and additional analysis comparing patients who used these drugs 24 hours before surgery with those who did not use antihypertensive drugs in those 24 hours****.***
